# Regorefenib induces extrinsic/intrinsic apoptosis and inhibits MAPK/NF‐κB‐modulated tumor progression in bladder cancer in vitro and in vivo

**DOI:** 10.1002/tox.22734

**Published:** 2019-02-25

**Authors:** Chih‐Hung Chiang, Jing‐Gung Chung, Fei‐Ting Hsu

**Affiliations:** ^1^ Department of Urology Medical Research and Education, Taipei Veterans General Hospital Yuan‐Shan/Su‐Ao Branch, Yilan Taiwan; ^2^ Department of Nursing Cardinal Tien Junior College of Healthcare and Management New Taipei City Taiwan; ^3^ Department of Urology National Taiwan University Hospital Taipei Taiwan; ^4^ Department of Biological Science and Technology China Medical University Taichung Taiwan; ^5^ Department of Biotechnology Asia University Taichung Taiwan

**Keywords:** apoptosis, bladder cancer, MAPK, NF‐κB, regorafenib

## Abstract

The aim of the present study is to investigate anticancer effect and mechanism of regorafenib in bladder cancer *in vitro* and *in vivo*. Human bladder cancer TSGH 8301 cells were treated with regorafenib, NF‐κB, AKT, or mitogen‐activated protein kinase (MAPK) inhibitors for different time. The changes of cell viability, NF‐κB activation, apoptotic signaling transduction, and expression of tumor progression‐associated proteins were evaluated with MTT, NF‐κB reporter gene assay, flow cytometry, and Western blotting assay. TSGH 8301 tumor bearing mice were established and treated with vehicle (140 μL of 0.1% DMSO) or regorafenib (10 mg/kg/day by gavage) for 15 days. The changes of tumor volume, body weight, NF‐κB activation, MAPK activation, and tumor progression‐associated proteins (MMP‐9, XIAP, VEGF, and Cyclin‐D1) after regorafenib treatment were evaluated with digital caliper, digital weight, and *ex vivo* Western blotting assay. Our results demonstrated NF‐κB activation and protein levels of MMP‐9, XIAP, VEGF, and Cyclin‐D1 were significantly reduced by NF‐κB (QNZ), ERK (PD98059), and P38 (SB203580) inhibitors. Regorafenib also significantly induced extrinsic and intrinsic apoptotic signaling transduction in bladder cancer *in vitro*. In addition, regorafenib significantly inhibited tumor growth, NF‐κB, p38, ERK activation and expression of tumor progression‐associated proteins in bladder cancer *in vitro* and *in vivo*. Taken together, these results proved that regorafenib not only induced apoptosis through extrinsic and intrinsic pathways and but suppressed MAPK/ NF‐κB‐modulated tumor progression in bladder cancer.

## 1 INTRODUCTION

Many epidemiological studies presented various environmental risk factors such as fungicide, tobacco, metals and motor vehicle exhaust, and occupational exposure to aromatic amines are associated with development of bladder cancer.^1^ Carcinogens‐induced genetic alteration result in conversion of proto‐oncogenes to oncogenes and silence of tumor suppressor genes. High expression of oncogenes and inactivation of tumor suppressor genes cause hyperactivation of signaling pathways involved in cell growth, survival, angiogenesis, and metastasis leading to cancer formation and progression.^2^, ^3^, ^4^

Impact of genetic alterations on dysregulated signaling transduction has been found in muscle invasive bladder cancers (MIBCs) and non‐MIBCs (NMIBCs).^5^ The p53 and RB (retinoblastoma) tumor suppressor pathway which controls restriction of cell cycle progression is altered and negatively regulated by mutation of tumor suppressor genes and expression of oncogenes.^6^ Hyperactivation of Ras/mitogen‐activated protein kinase (MAPK) pathway contributes to tumor progression. Overexpression of Ras/MAPK pathway is modulated by Ras, HER2 and HER3 mutation, and EGFR amplification in bladder cancer.

Ras/MAPK pathway has been targeted by tyrosine kinase inhibitors (TKIs) in clinical trials.^4^, ^6^, ^7^, ^8^ Regorafenib (Stivarga) is an oral multiple kinase inhibitor which targets several critical signaling molecules including angiogenic, stromal, oncogenic receptor tyrosine kinases and already approved for treatment of patients with colorectal cancer, gastrointestinal stromal tumors, and hepatocellular carcinoma (HCC).^9^, ^10^ Regorafenib has been indicated to diminish tumor progression through suppression of MAPK/extracellular signal‐regulated kinase (ERK) activation in HCC *in vitro* and *in vivo* 11, 12. In previous study presented regorafenib induces apoptosis and inhibits metastatic potential in bladder cancer.^13^ However, effect of regorafenib on tumor progression in bladder cancer is ambiguous. Therefore, the aim of the present study was to verify anti‐cancer effect and mechanism of regorafenib in bladder cancer *in vitro* and *in vivo*.

## 2 METHODS

### 2.1 Chemical reagents and antibodies

Regorafenib was kindly provided by Bayer Corporation (Whippany, NJ, USA). We purchased MTT (3‐[4,5‐Dimethylthiazol‐2‐yl]‐2,5‐Diphenyltetrazolium Bromide), RNase and dimethyl sulfoxide (DMSO) from Sigma Chemical Co. (St. Louis, MO, USA). For cell culture reagents, RPMI 1640, fetal bovine serum (FBS), l‐glutamine and penicillin‐streptomycin were all obtained from GIBCO/Invitrogen Life Technologies (Carlsbad, CA, USA). NF‐κB‐luciferrase2 vector (pNF‐κB/luc2) and d‐luciferin were obtained from Promega (Madison, WI, USA) and Caliper Life Science (Hopkinton, MA, USA), respectively. NF‐κB inhibitor 4‐*N*‐[2‐(4‐phenoxyphenyl)ethyl]quinazoline‐4,6‐diamine (QNZ), AKT inhibitor LY294002, c‐Jun N‐terminal kinase (JNK) inhibitor SP600125, P38 inhibitor SB203580 and extracellular signal‐regulated kinase (ERK) inhibitor PD98059 were purchased from Selleckchem (Houston, TX, USA). Hygromycin B was purchased from Santa Cruz Biotechnology. The primary antibodies were purchased from different companies as described as follows: MMP‐9 (EMD Millipore, Billerica, MA, USA), VEGF (EMD Millipore), C‐FLIP (Cell signaling Technology, Danvers, MA, USA), Phospho‐p44/42 MAPK (Erk1/2) (Cell signaling), Phospho‐p38 MAPK (Thr180/Tyr182) (Cell signaling), p38 MAPK (Cell signaling), tERK (Santa Cruz, CA, USA), Phospho‐p65 NF‐κB (Ser276) (Signalway Antibody LLC, MD, USA), p65 NF‐κB antibody (Abcam plc, Canary Wharf, London, UK), XIAP (Thermo Fisher Scientific, Fremont, CA, USA), Cyclin D1 (Thermo Fisher Scientific), β‐actin (Santa Cruz), TBP (Abcam plc.) for Western blotting evaluation. Secondary antibodies were purchased from Jackson ImmunoResearch (West Grove, PA, USA). CaspGlow fluorescein active Caspase‐3 and ‐8 staining kit was acquired from BioVision (Milpitas, CA, USA). Annexin V‐FITC apoptosis detection kit was purchased from Vazyme Biotech Co. Lt (Nanjing City, China). Anti‐FAS‐FITC, anti‐FASL‐PE and cleaved PARP1‐FITC were purchased from Thermo Fisher Scientific.

### 2.2 Culture of TSGH cells

TSGH 8301 human bladder carcinoma cell line was obtained from Professor Jing‐Gung Chung's lab, China Medical University, and routinely tested for mycoplasma contamination.^14^, ^15^ TSGH 8301 cells were seeded on 10 cm tissue culture plate with RPMI‐1640 medium and containing 10% FBS, 2 mM l‐glutamine, and 1% penicillin–streptomycin (100 U mL^−1^ penicillin and 100 μg/mL streptomycin) and grown at 37°C under a humidified 5% CO_2_ and one atmosphere.

### 2.3 Identify the cytotoxicity effect of regorafenib by MTT assay

MTT assay was done to evaluate the cell viability after regorafenib treatment in culture system. Thirty thousand of TSGH 8301 cells were placed in 96‐well plates overnight and then treated with various dose of regorafenib (0–40 μM) for 24 and 48 h. Medium were removed by suction machine, replaced by MTT solution (0.5 mg/mL) and maintained in incubator for 4 h at 37°C. DMSO was finally used to dissolve the insoluble purple formazan product into a colored solution and then prepared for measurement. The absorbance of this colored solution can be quantified by measuring at a certain wavelength (570 nm) by a spectrophotometer (Tecan Group Ltd., Männedorf, Switzerland).^11^

### 2.4 Established NF‐κB reporter gene system and monitor the effect of regorafenib on NF‐κB activation

Plating 1 × 10^6^ TSGH 8301 cells in a 100‐mm culture dishes in 10 mL of RPMI‐1640 medium overnight and achieved the desired density of 80% confluence before transfection. Cells were transfected with NF‐κB luciferase reporter plasmid (pNF‐κB/*luc2*) (Promega, Madison, WI, USA) by jetPEI transfection reagent (Illkirch, Bas‐Rhin, France) and then selected with 200 μg/mL of hygromycin B for 2 weeks as described previously. Stable clones which constitutively express function of NF‐κB reporter gene were rename as TSGH/NF‐κB‐*luc2* cells and used for NF‐κB reporter gene assay.^11^ For NF‐κB luciferase assay, 3 × 10^4^ TSGH/NF‐κB‐*luc2* cells were seeded in 96 wells overnight before regorafenib (0–40 μM) or different inhibitor (0.5 μM NF‐κB inhibitor, 10 μM ERK inhibitor, 10 μM p38 inhibitor, 10 μM AKT inhibitor, 10 μM JNK inhibitor) treatment for 48 h. Luciferase reagent, d‐luciferin solution (500 μM d‐luciferin in 100 μL PBS), was added into each well for 10 min incubation. Photon signal (p/s/cm^2^/sr) of luc2 reporter gene was acquired for 3 min using the IVIS 200 Imaging System. The expression of NF‐κB was quantified by Living Image software (Version 2.20; Xenogen, Alameda, CA, USA) and normalized with viable cells number.^11^

### 2.5 Evaluate the suppression effect of regorafenib on tumor progression proteins by Western blot

About 3 × 10^6^ cells TSGH 8301 cells were incubated in a 100‐mm culture dishes overnight and then treated with 0, 15, or 30 μM regorafenib for 48 h, respectively. Total cells and tumor tissues were collected and gently re‐suspended in lysis buffer [50 mM Tris–HCl (pH 8.0), 120 mM NaCl, 0.5% Nonide P‐40] for sonication and centrifuged as described previously and supernatant was used for measuring total protein by the Pierce BCA protein assay kit (Thermo Fisher Scientific) with bovine serum albumin (BSA) as the standard.^16^ Total protein was electrophoresed on SDS polyacrylamide gels and then electrotransfered onto PVDF membrane (EMD Millipore), washed and incubated with primary antibodies (anti‐MMP‐9, ‐XIAP, ‐VEGF, ‐CyclinD1, ‐p‐ERK, ‐t‐ERK, ‐p65‐NF‐κB, ‐NF‐κB, ‐p‐p38, ‐t‐p38, and ‐β‐actin). After washed, the membranes were incubated with HRP conjugated anti‐rabbit IgG or anti‐mouse IgG. Immunoreactive proteins were visualized and detected by Immobilon Western Chemiluminescent HRP Substrate (Pierce, Rockford, IL, USA). The band intensities of protein were quantified using the Image J (version 1.50; National Institutes of Health, Bethesda, MD, USA).

### 2.6 Evaluate regorafenib‐induced apoptotic signaling by flow cytometry

TSGH 8301 cells (5 × 10^5^) were seeded six‐well plates overnight and then were incubated with 0, 15, and 30 μM of regorafenib for 48 h. For cell cycle phase analysis, cells were harvested, fixed by 70% ethanol overnight and stained with PI (40 μg/mL).^17^ For apoptosis activity and mitochondria potential loss, cells were single stained by caspase‐3, caspase‐8, DIOC_6_, respectively. Staining procedure has been describing in detail in previous studies.^13^, ^18^ In addition, for double stain, cells were harvested and stained with Annexin V/PI or FAS/FASL double staining for total apoptotic cell death analysis.^19^ For PARP1 analysis, cells were fixed with 4% formaldehyde fixation buffer for 15 min. Cells were then washed and refreshed with methanol permeabilization buffer (a final concentration 90% ice‐cold methanol) overnight. On the second day, washed cells by centrifugation in excess 1X PBS to remove methanol and resuspended cells in 100 μL of diluted antibody conjugate [1:50 = 2 μL PARP1 antibody:98 μL incubation buffer (0.5% Bovine Serum Albumin buffer)] for 1 h.^20^ After staining by different reagents, the change of subG1 population, caspase‐3, caspase‐8, DIOC_6_, Annexin V/PI, FAS/FASL, PARP1 was detected by flow cytometry, respectively, (BD Biosciences, FACS Calibur, San Jose, CA, USA). Five repeated results were all analyzed by FlowJo software (version 7.6.1; FlowJo LLC, Ashland, OR, USA).

### 2.7 Detect tumor growth inhibition of regorafenib in vivo

All animal care and experimental procedures were executed by with the guidelines for the use of laboratory animals, and approved by the Institutional Animal Care and Use Committee in China Medical University (No: CMU IACUC‐2019‐018). Ten million of TSGH 8301 human bladder carcinoma cells (per mouse) were mixed with an equal volume of BD Matrigel (Becton, Dickinson and Company, Franklin Lakes, NJ, USA) and subcutaneous injected into mice right flank (*N* = 20).^15^ When the tumor size reached approximately 100 mm^3^, the mice were randomly divided into two groups and received the following treatments: CTRL (0.1% DMSO) and regorafenib (10 mg/kg). Tumor volumes were calculated from the following formula: tumor volume = length × width × thickness × 0.523.^12^ Body weight was measured by digital weight every 3 days. Mice were sacrificed and tumors were finally extracted, measured by digital weight on day 15 and lysed for *ex vivo* Western blot.

### 2.8 Statistical analysis between different treatment groups

Results are presented as mean ± SD. The significant difference between regorafenib‐treated and non‐treated control groups were analyzed by Student's *t*‐test. *P* < 0.05 and *P* < 0.01 were both defined as an indication of statistical significance.

## 3 RESULTS

### 3.1 Regorafenib enhanced bladder cancer cells cytotoxicity through blockage of p38 MAPK‐ and ERK‐mediated NF‐κB pathway

**Figure 1:**
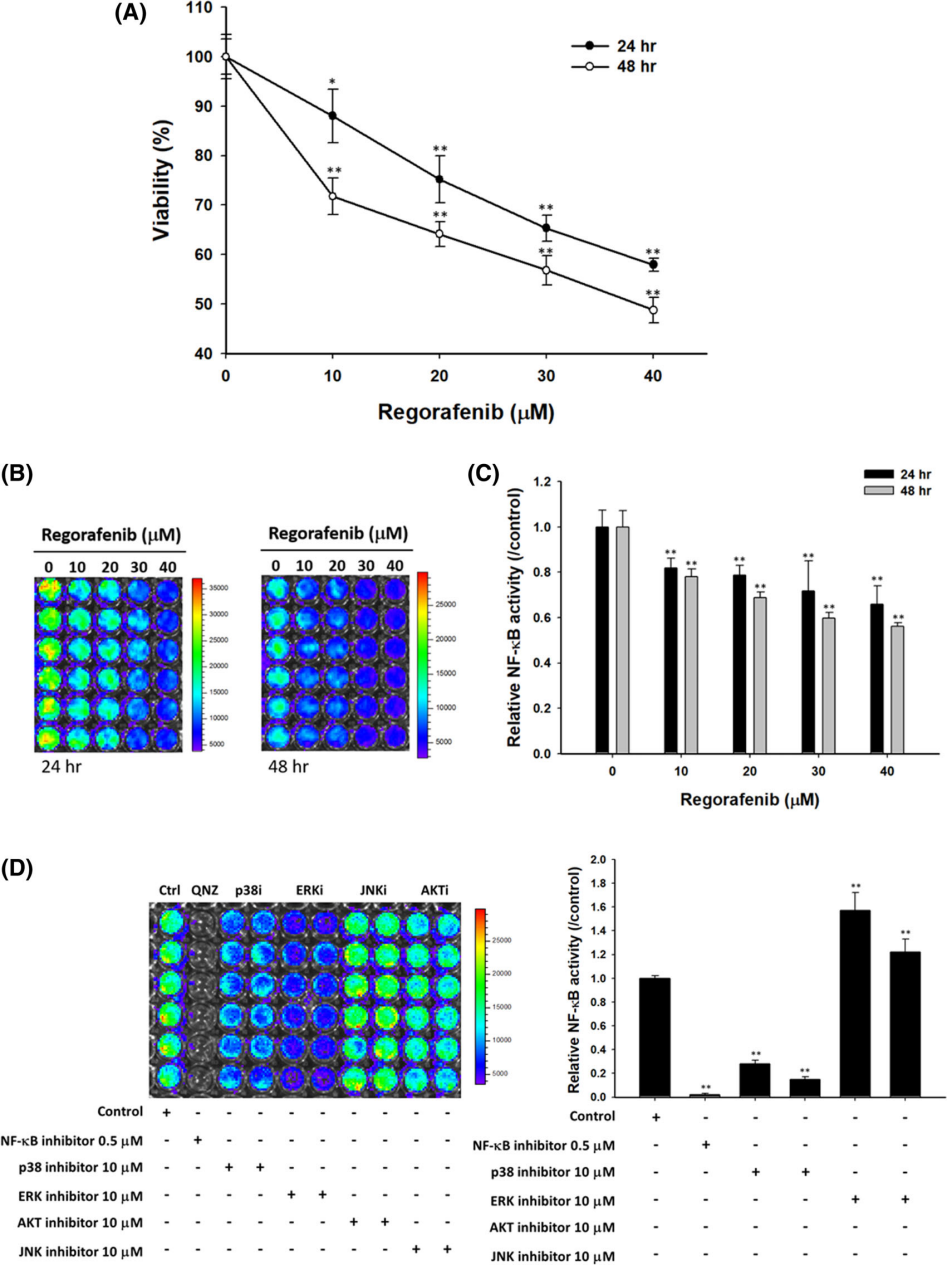
Regorafenib‐reduced viable cells number via inhibition of p38 MAPK and ERK mediated NF‐κB pathway. (A) TSGH 8301 cells were prepared for total cell viability measurements, which were treated with 0, 10, 20, 30, 40 μM of regorafenib for 24 or 48 h. (B, C) Cells were measured for the activation of NF‐κB after regorafenib treated by reporter gene assay for 24 or 48 h. (D) The activation of NF‐κB were detected by IVIS after different kind of inhibitor treatment under 10 μM dosage for 48 h, including NF‐κB (0.5 μM), p38, ERK, JNK and AKT inhibitor. (QNZ= NF‐κB inhibitor;p38i= p38 inhibitor; ERKi= ERK inhibitor; JNKi= JNK inhibitor and AKTi= AKT inhibitor) **P < 0.01, significant difference between treated groups and the control as analyzed by Student's *t‐*test [Color figure can be viewed at wileyonlinelibrary.com]

In Figure 1A, regorafenib markedly enhanced cytotoxicity of TSGH 8301 cells by dose and time. We further investigated whether regofenib may affect the activation of NF‐κB by reporter gene assay. The activation of NF‐κB was markedly suppressed by regorafenib with time‐ and dose‐dependent manner (Figure 1B). Quantification results, Figure 1C, demonstrated 20–50 suppression percentage of NF‐κB activity by regorafenib. Then, we further investigated the upstream signaling transduction of regorafenib on TSGH8301 cells by treated with different inhibitors, such as NF‐κB inhibitor, p38 MAPK inhibitor, ERK inhibitor, JNK inhibitor and AKT inhibitor. Our results suggested that only p38 MAPK inhibitor and ERK inhibitor showed similar NF‐κB suppression efficacy as NF‐κB inhibitor (Figure 1D). In sum, these results indicated that the toxicity effect of regorafenib was mediated by the inhibition of p38 MAPK‐ and ERK‐mediated NF‐κB pathway.

### 3.2 Regorafenib inhibited tumor progression‐related gene expression through blockage of p38 MAPK and ERK signaling transduction

**Figure 2:**
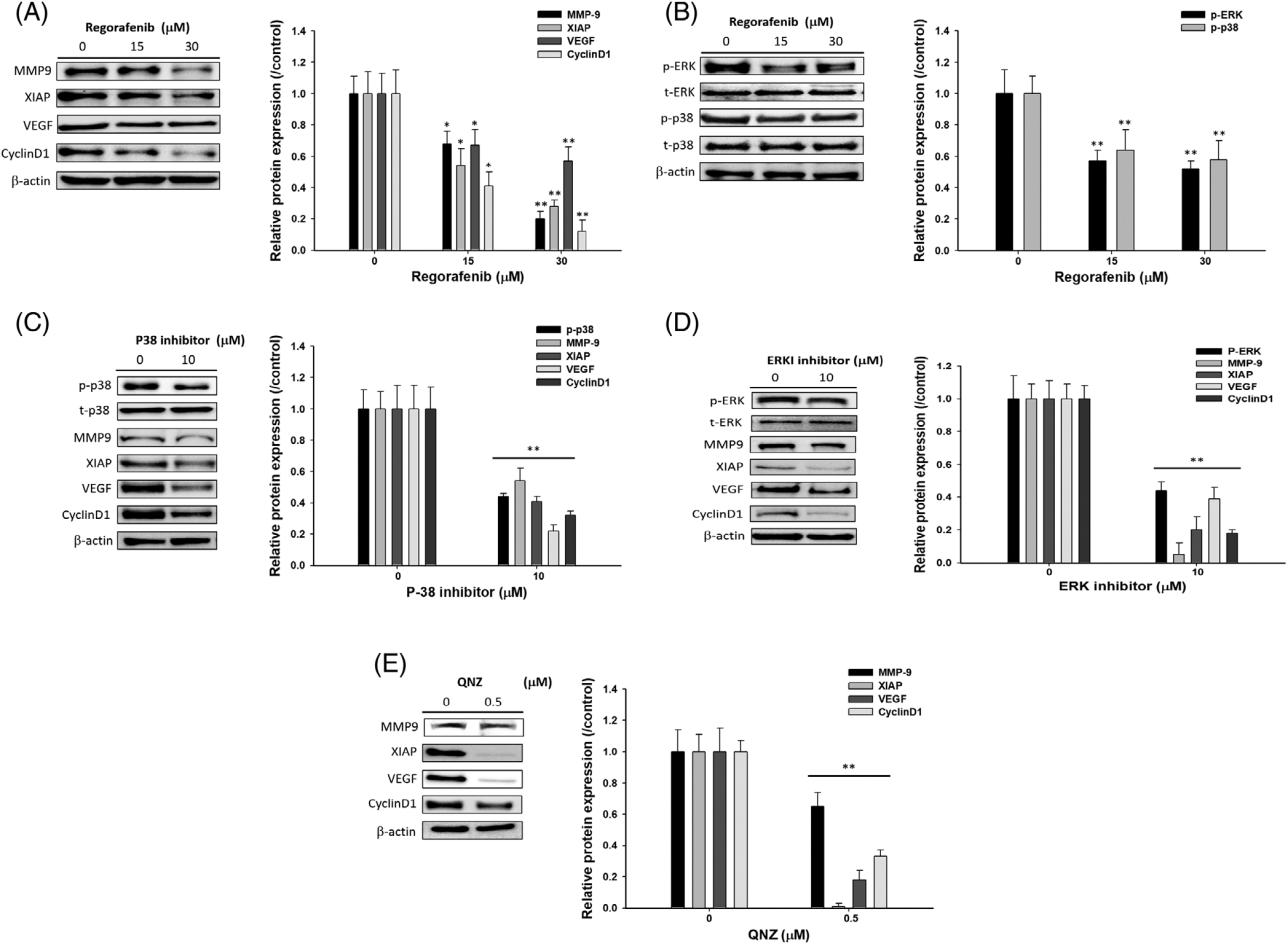
Regorafenib affected tumor progression‐associated protein expression in TSGH 8301 cells. TSGH 8301 cells were treated with regorafenib (15 and 30 μM), p38 inhibitor (10 μM), ERK inhibitor (10 μM) and QNZ (10 μM) for 48 h. Cells were collected and total protein was determined for SDS‐PAGE gel electrophoresis, as described in Section 2. The levels of MMP‐9, XIAP, VEGF, and CyclinD1 (A); p‐p38 and p‐ERK1/2 (B) after regorafenib treatment, p38 inhibitor treatment (C), ERK inhibitor treatment (D) or QNZ treatment (E) were estimated by Western blotting. **P < 0.01, significant difference between regorafenib‐treated groups and the control as analyzed by Student's *t*‐test

After we confirmed the toxicity and NF‐κB inhibition ability of regorafenib, we further investigate downstream molecules that mediated by NF‐κB. Here, we found that invasion related MMP‐9, proliferation related XIAP and CyclinD1, angiogenesis‐related VEGF proteins were all decreased by regorafenib (Figure 2A). Moreover, the phosphorylation of p38 MAPK and ERK were both reduced by regorafenib (Figure 2B). To inveterate the tumor progression inhibitory effect of regorafenib was through the modulation of p38 MAPK‐ and ERK‐mediated NF‐κB signaling, we performed with Western blot for tumor progression related proteins evaluation by treated cells with p38 MAPK and ERK inhibitors. As showed in Figure 2C, all NF‐κB mediated tumor progression related markers were decreased by p38 MAPK inhibitor. In ERK inhibitor treated groups, we found similar inhibition result as p38 MAPK inhibitor (Figure 2D). We finally validated whether NF‐κB inhibitor may demonstrate compatible results as Figure 2C,D. Figure 2E specified that the suppression of NF‐κB was certainty reduced the expression of MMP9, XIAP, VEGF, CyclinD1. In sum, regorafeinb may reduce tumor progression through diminish of p38 MAPK‐ and ERK‐mediated NF‐κB pathway.

### 3.3 Regorafenib‐induced mitochondria dependent apoptosis and extrinsic apoptosis of bladder cancer cells

**Figure 3:**
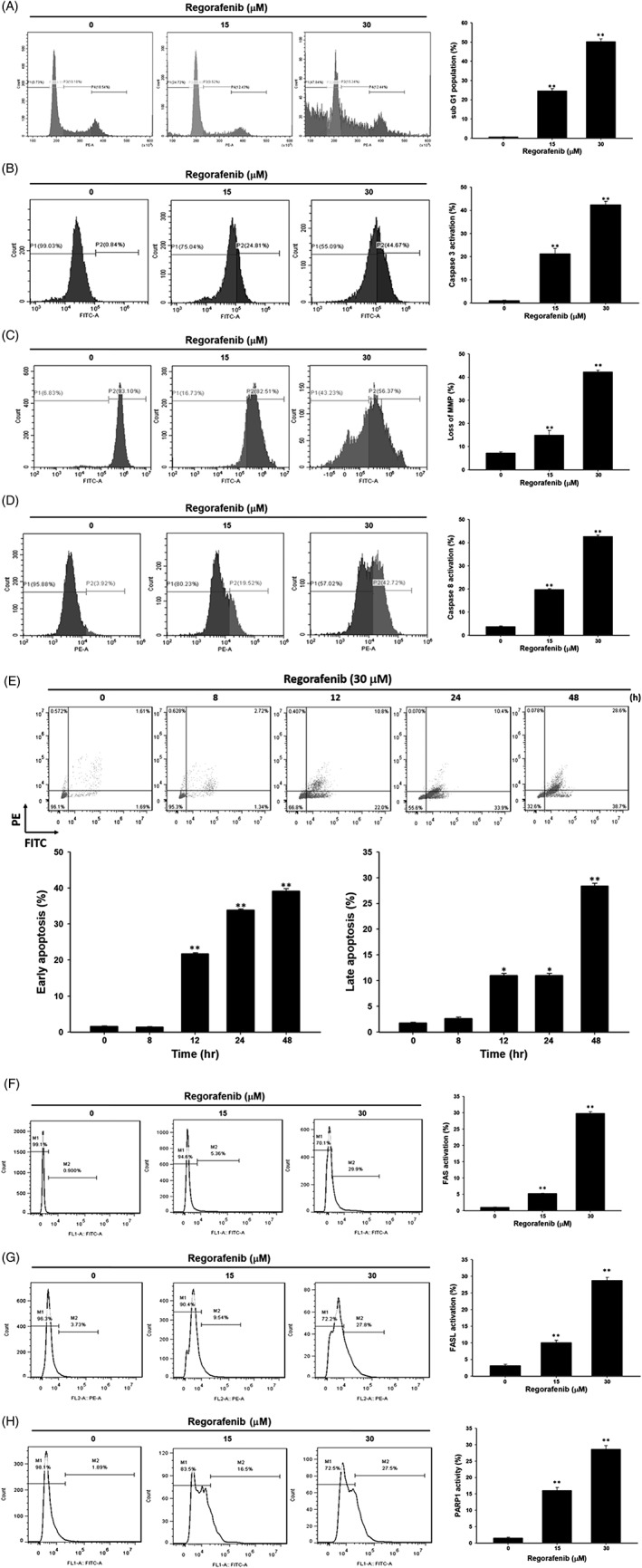
Regorafenib affected the levels of subG1 phase, caspase‐3 activities, mitochondrial membrane potential (MMP), caspase‐8 activities, Fas activities, FasL activities and cleavage PARP1 activities in TSGH8301 cells. TSGH 8301 cells were incubated with 15 μM or 30 μM of regorafenib for 48 h. Harvested for (A) cell cycle analysis (B) caspase‐3 activities measurement (C) MMP measurement (D) caspase‐8 activities measurement (E) AnnexinV/PI (F) Fas activities measurement (G) FasL activities measurement (H) PARP1 activities measurement using flow cytometric assay. *P < 0.05 and **P < 0.01, significant difference between regorafenib‐treated groups and the control as analyzed by Student's *t*‐test

In cell cycle process, subG1 population has been recognized as apoptosis phase of cells. In Figure 3A, the population of subG1 was significantly increased to 20%–50% by regorafenib, which represent the apoptosis effect was increased. Caspase‐3 was known as an inevitable marker in the process of apoptosis. We found the obvious induction of caspase‐3 activity in regorafenib treated cells (Figure 3B). For intrinsic apoptosis marker, mitochondria membrane potential was noticeably loss after regorafenib administrated (Figure 3C). Additionally, extrinsic apoptosis marker caspase‐8 was induced by regorafenib (Figure 3D). In annexin V and PI double staining results, we found that the early and late apoptosis were both induced around 20%–40% in TSGH 8301 cells by time (Figure 3E). Finally, we further confirmed whether the death receptor and ligand were both modulated by regorafeinb. As showed in Figure 3F,G, the activation of Fas and FasL in regroafenib treated group was found. Furthermore, regorafenib not only enhanced extrinsic and intrinsic apoptosis of bladder cancer cells but also induced the cleavage of PARP1 (Figure 3H), which also demonstrated as an apoptosis marker.

### 3.4 Regorafenib suppressed the growth and expression of tumor progression factors in bladder cancer in vivo

**Figure 4:**
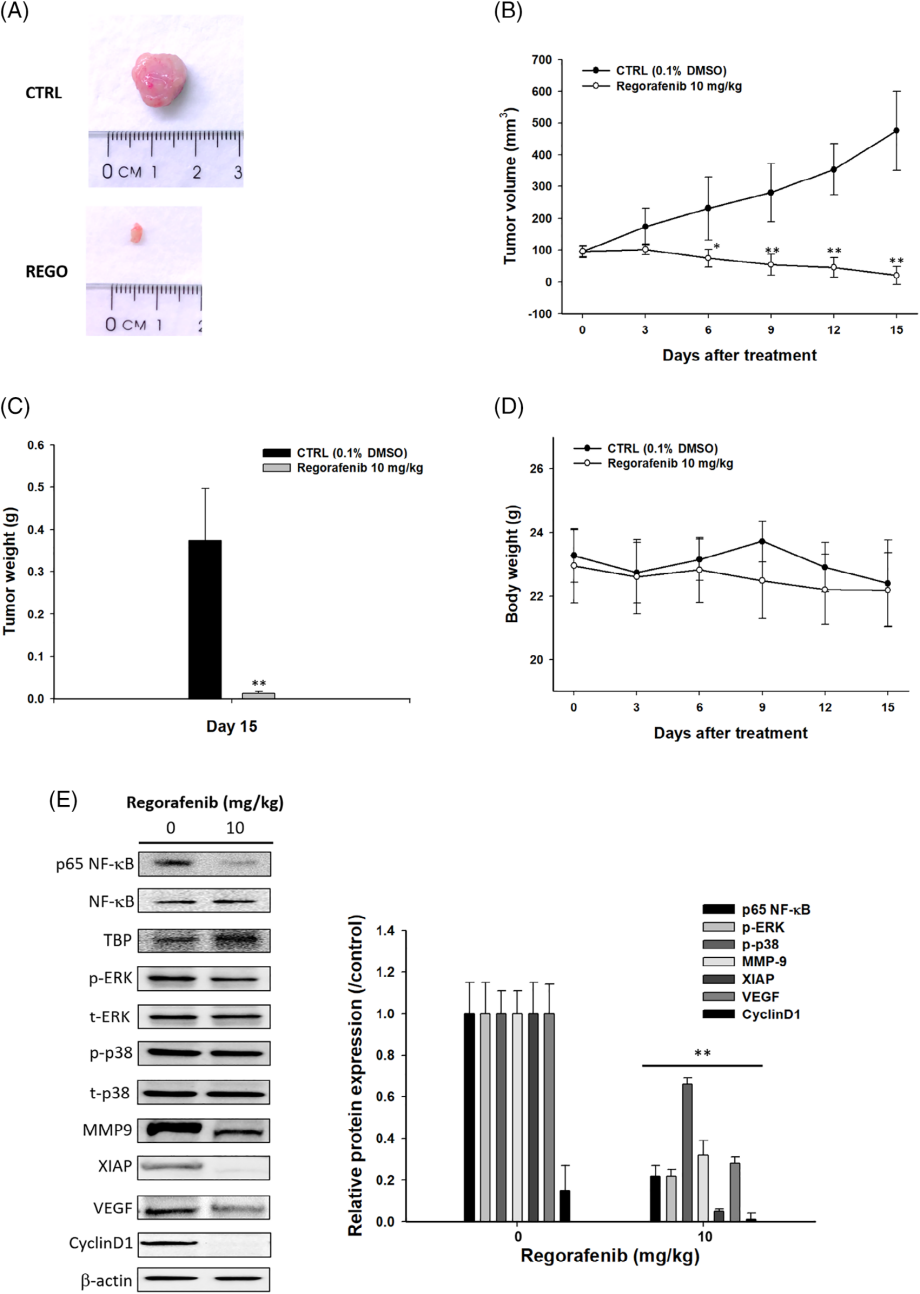
Regorafenib affected TSGH8301 cell bearing xenograft nude mice. Twenty male BALB/c nude mice were subcutaneously (s.c.) injected with TSGH 8301 cells (1 × 10^7^ cells/mice) into the right flanks on mice for 15 days. After the tumors volume reached to 100 mm^3^, and then regorafenib treatment was started. The total 20 animals were divided into two groups of 10 animals each. (A) After treatment, the tumor of each animal was observed and photographed. The tumor volumes (B) and tumors weights (C), and body weight (D) were measured. (E) The protein level within tumor tissue were evaluated by Western blot [Color figure can be viewed at wileyonlinelibrary.com]

Figure 4A indicated that one representative animal tumor on each treatment of mice. Tumor size was markedly decreased after regorafenib treatment. After animals were treated, body weights and tumor volumes were measured once per 3 days. Figure 4B indicated that regorafenib significantly reduced tumor volume when compared to non‐treated control groups. Figure 4C indicated the tumor weight from each treatment and demonstrated that regorafenib significantly reduced of tumor weight. Figure 4D indicated that regorafenib did not significantly affect the body weights when compared to control groups. After tumors were removed from each group of animals, proteins were extracted, assayed by Western blotting and results were shown in Figure 4E. Figure 4E indicated that regorafenib significantly decreased MMP‐9, XIAP, VEGF, and CyclinD1 when compared to non‐treated control groups. Moreover, as showed in Figure 4E, regorafenib inhibited tumor growth and tumor progression through dephosphorylating of p38 MAPK, ERK, NF‐κB signaling transduction.

## 4 DISCUSSION

Death receptor Fas (CD95)/Fas ligand (FasL) interaction initiates extrinsic apoptotic signaling pathway through triggering caspase‐8 activation.^21^ Loss of mitochondrial membrane potential promotes release of pro‐apoptotic proteins from mitochondria leading to intrinsic apoptosis.^22^ Caspase‐3‐modulated deoxyribonucleic acid (DNA) fragmentation and cleavage of poly (ADP‐ribose) polymerase 1 (PARP1) can be activated by extrinsic and intrinsic apoptotic pathways.^23^ Decreased expression of Fas and Caspase‐3 is associated with poor prognosis in patients with bladder cancer.^21^, ^24^ PARP1, an ADP‐ribosylating enzyme, participates in repair of various forms DNA damage and maintains chromatin remodeling.^25^ Overexpression of PARP1 has been found in high stage cancers.^26^, ^27^ Liu et al.^28^ presented PARP1 inhibitor not only induces DNA double‐strand breaks (DSB) but also sensitizes bladder cancer cells to radiation. We found regorafenib significantly induces apoptosis and increases activation of Fas/FasL, Caspase‐8, ‐3, loss of ΔΨm, and cleavage of PARP1 in bladder cancer TSGH 8301 cells.

Nuclear factor‐κB (NF‐κB) family of transcription factors are composed of five subunits including RelA (p65), RelB, c‐Rel, NF‐κB1 (p105/p50), and NFκB2 (p100/p52). NF‐κB p50/p65 heterodimer is critical mediator for tumorigenesis and cancer progression. A number of tumor progression‐associated proteins which modulate proliferation, anti‐apoptosis, angiogenesis, and metastasis are encoded by NF‐κB target genes and upregulated with active NF‐κB signaling in bladder cancer.^29^, ^30^ Constitutive NF‐κB activation was observed in high grade bladder cancer.^31^ Many studies indicated inhibition of NF‐κB activation suppresses tumor growth, anti‐apoptosis, angiogenesis, and metastatic potential in bladder cancer in vitro and in vivo.^32^, ^33^ In our results demonstrated NF‐κB activation and expression of tumor progression‐associated proteins were significantly decreased by QNZ (NF‐κB inhibitor) in TSGH‐8301 cells. We also found regorafenib significantly reduces tumor growth, NF‐κB activation, and protein levels of tumor progression‐associated proteins (MMP‐9, XIAP, VEGF, and Cyclin‐D1) in bladder cancer *in vitro* and *in vivo*.

Mitogen‐activated protein kinase family which include ERK, p38, and c‐Jun NH2‐terminal kinase (JNK) modulate tumor progression through upregulation of downstream kinases and transcription factors.^34^ NF‐κB activation may be regulated by AKT or MAPKs cascades.^11^ We used NF‐κB reporter gene assay to evaluate effect of AKT or MAPK inhibitors on NF‐κB activation in TSGH 8301 cells. The results presented both p38 (SB203580) and ERK inhibitor (PD98059) significantly inhibit NF‐κB activation in TSGH 8301 cells. Harb et al.^35^ found high expression of p38 as poor prognostic marker was correlated with high grade stage and distant metastasis in bladder cancer. ERK and p38 MAPK activate cell growth and invasive ability in bladder cancer cells.^36^, ^37^ In our results presented protein levels of MMP‐9, XIAP, VEGF, and Cyclin‐D1 were significantly reduced by PD98059 or SB203580 treatment, respectively. In addition, protein levels of phosphorylated ERK (p‐ERK) and p38 (p–p38) were diminished by regorafenib treatment.

**Figure 5:**
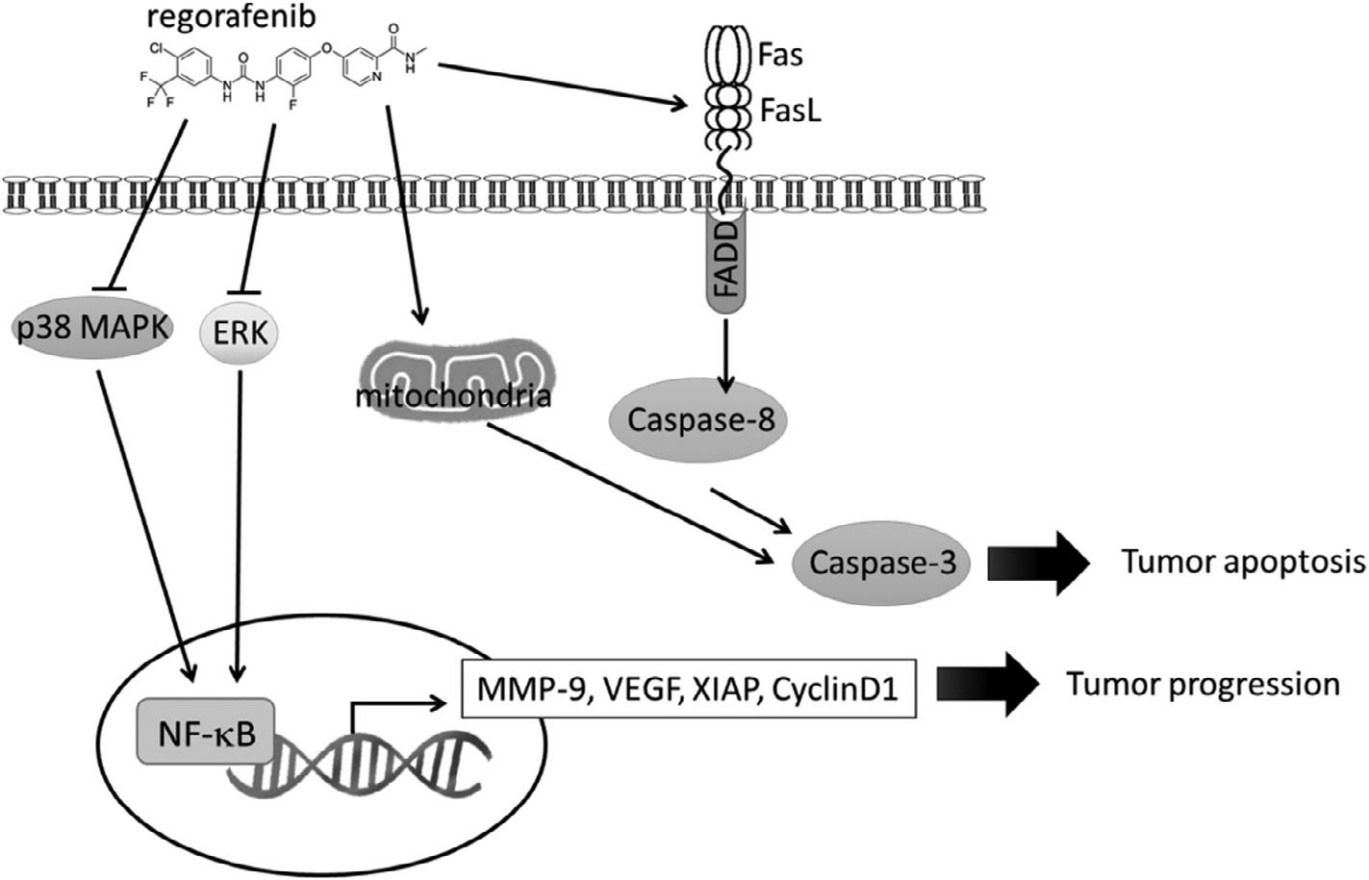
The possible signaling pathways for regorafenib inhibited tumor progression and induced apoptosis of TSGH 8301 human bladder cancer cells. Regorafenib can inhibit tumor progression related ability, such as cell proliferation, angiogenesis, cell migration and invasion in bladder TSGH 8301 cancer cells. Regorafenib inhibits the expressions of signaling molecules involved in p‐ERK, p‐38 MAPK and NF‐κB signaling pathways for leading to the inhibition of MMP‐9, VEGF, XIAP, and CyclinD1 in TSGH8301 cancer cells

In conclusion, regorafenib not only induces extrinsic/intrinsic apoptosis and inhibits MAPK/NF‐κB‐modulated tumor progression in bladder cancer *in vitro* and *in vivo* (Figure 5). We demonstrated regorafenib can be considered as a potential treatment, which offer therapeutic benefit for patients with bladder cancer.

## CONFLICT OF INTEREST

The authors do not have any conflicts of interest to disclose.
